# Colon Tumors in Enterotoxigenic Bacteroides fragilis (ETBF)-Colonized Mice Do Not Display a Unique Mutational Signature but Instead Possess Host-Dependent Alterations in the APC Gene

**DOI:** 10.1128/spectrum.01055-22

**Published:** 2022-05-19

**Authors:** Jawara Allen, Axel Rosendahl Huber, Cayetano Pleguezuelos-Manzano, Jens Puschhof, Shaoguang Wu, Xinqun Wu, Charelle Boot, Aurelia Saftien, Heather M. O’Hagan, Hao Wang, Ruben van Boxtel, Hans Clevers, Cynthia L. Sears

**Affiliations:** a Department of Medicine, Johns Hopkins University School of Medicine, Baltimore, Maryland, USA; b Medical Sciences Program, Indiana University School of Medicine, Bloomington, Indiana, USA; c Indiana University Melvin and Bren Simon Comprehensive Cancer Center, Indianapolis, Indiana, USA; d Department of Oncology, Johns Hopkins Medicine Institutions, Baltimore, Maryland, USA; e Bloomberg-Kimmel Institute for Cancer Immunotherapy, Sidney Kimmel Comprehensive Cancer Center, Johns Hopkins Medicine Institutions, Baltimore, Maryland, USA; f Hubrecht Institute, Royal Netherlands Academy of Arts and Sciences (KNAW) and UMC Utrecht, Utrecht, The Netherlands; g Oncode Institute, Utrecht, The Netherlands; h The Princess Máxima Center for Pediatric Oncology, Utrecht, The Netherlands; i Division of Biostatistics and Bioinformatics, Sidney Kimmel Comprehensive Cancer Center, Johns Hopkins Medicine Institutions, Baltimore, Maryland, USA; j Cell, Molecular and Cancer Biology Program, Indiana University School of Medicine, Bloomington, Indiana, USA; k Sidney Kimmel Comprehensive Cancer Center, Johns Hopkins Medicine Institutions, Baltimore, Maryland, USA; University of Nebraska-Lincoln

**Keywords:** *Bacteroides*, cancer, colorectal cancer, genomics, microbes, mutational studies

## Abstract

Enterotoxigenic Bacteroides fragilis (ETBF) is consistently found at higher frequency in individuals with sporadic and hereditary colorectal cancer (CRC) and induces tumorigenesis in several mouse models of CRC. However, whether specific mutations induced by ETBF lead to colon tumor formation has not been investigated. To determine if ETBF-induced mutations impact the *Apc* gene, and other tumor suppressors or proto-oncogenes, we performed whole-exome sequencing and whole-genome sequencing on tumors isolated after ETBF and sham colonization of *Apc*^min/+^ and *Apc*^min/+^*Msh2^fl/fl^*VC mice, as well as whole-genome sequencing of organoids cocultured with ETBF. Our results indicate that ETBF-induced tumor formation results from loss of heterozygosity (LOH) of *Apc*, unless the mismatch repair system is disrupted, in which case, tumor formation results from new acquisition of protein-truncating mutations in *Apc*. In contrast to polyketide synthase-positive Escherichia coli (*pks*+ E. coli), ETBF does not produce a unique mutational signature; instead, ETBF-induced tumors arise from errors in DNA mismatch repair and homologous recombination DNA damage repair, established pathways of tumor formation in the colon, and the same genetic mechanism accounting for sham tumors in these mouse models. Our analysis informs how this procarcinogenic bacterium may promote tumor formation in individuals with inherited predispositions to CRC, such as Lynch syndrome or familial adenomatous polyposis (FAP).

**IMPORTANCE** Many studies have shown that microbiome composition in both the mucosa and the stool differs in individuals with sporadic and hereditary colorectal cancer (CRC). Both human and mouse models have established a strong association between particular microbes and colon tumor induction. However, the genetic mechanisms underlying putative microbe-induced colon tumor formation are not well established. In this paper, we applied whole-exome sequencing and whole-genome sequencing to investigate the impact of ETBF-induced genetic changes on tumor formation. Additionally, we performed whole-genome sequencing of human colon organoids exposed to ETBF to validate the mutational patterns seen in our mouse models and begin to understand their relevance in human colon epithelial cells. The results of this study highlight the importance of ETBF colonization in the development of sporadic CRC and in individuals with hereditary tumor conditions, such as Lynch syndrome and familial adenomatous polyposis (FAP).

## INTRODUCTION

Rates of human colorectal cancer (CRC) are increasing in younger individuals (1), and CRC remains the second most frequent cause of cancer-related deaths globally (2). Multiple risk factors contribute to the onset of CRC (e.g., colon inflammation, obesity, diabetes), each now known to modify the human colon microbiome. Moreover, many studies have shown that microbiome composition and structure in the mucosa and/or stool differ in individuals with CRC (3–8), and in some cases, this difference is even seen when comparing tumor to flanking normal tissue (4, 6, 9–21). While it remains unknown whether these microbial changes directly induce tumor formation in the human colon, individual bacterial strains and communities of bacteria have been shown to directly promote colon tumor formation in several mouse models, many of which utilize mutations in *Apc* and/or *Msh2* (6, 10–19, 22, 23).

The tumor suppressor gene *APC* is mutated in the majority of sporadic CRC cases in humans (24), and a germ line mutation in *APC* leads to a hereditary tumor syndrome known as familial adenomatous polyposis (FAP). In cases of both sporadic and hereditary CRC, two “hits” in *APC* trigger colon tumor formation. These “hits” can result from protein-truncating mutations in *APC*, loss of heterozygosity (LOH) of *APC*, or epigenetic changes, such as methylation, that silence *APC* expression (24–26). Mice with various alterations in *Apc* have long been used to investigate CRC (27). These models generally produce many polyps in the small bowel but very few in the colon (27).

In contrast, while *MSH2* is inactivated in only 10% to 20% of sporadic cases of CRC (28–30), germ line mutations in *MSH2* are one of the most common causes of Lynch syndrome, a hereditary cancer syndrome in which individuals are at increased risk of developing several cancers, including colorectal, endometrial, urinary tract, small bowel, ovarian, stomach, and biliary tract (31). Mouse models with *Msh2* mutations have been utilized to better understand the pathophysiology encompassing mismatch repair-mediated tumor formation. To study the contribution of *Msh2* to CRC specifically, some of these models combine an intestine-specific *Msh2* mutation driven by villin-cre with an *Apc* mutation. In these mice (*Apc*^min/+^*Msh2^fl/fl^*VC mice), tumors develop copiously in the small bowel but sparingly in the colon (32).

Enterotoxigenic Bacteroides fragilis (ETBF), an anaerobic bacterium and common member of the human colon microbiota, is associated with human sporadic and hereditary CRC (10–12, 23) and induces colon tumors in mouse models (32, 33) as reviewed previously (34). When ETBF colonizes either *Apc*^min/+^ mice or *Apc*^min/+^*Msh2^fl/fl^*VC mice, the rate of tumor formation in the distal colon, but not the proximal colon or the small intestines, increases when compared to uninoculated (sham) mice or mice persistently colonized with nontoxigenic B. fragilis (33, 35, 36). Extensive histopathology and immunological analyses of mouse colons post ETBF-colonization have demonstrated that tumor formation in ETBF-colonized mice is accompanied by interleukin-17 (IL-17)-dominant colon inflammation (33, 35, 37), cleavage of the colon epithelial cell (CEC) adherens junctional protein E-cadherin (38), and activation of CEC signal transduction pathways including β-catenin/Tcf, NF-κB, and mitogen-activated protein kinase (MAPK) (22, 39–41). Additionally, Chung et al. (35) showed that ETBF colonization is uniform along the colonic axis (i.e., there is no ETBF colon bacterial gradient) after ETBF inoculation; instead, the distal localization of ETBF tumors is likely due to differential NF-κB activation by ETBF colonization in the distal versus proximal colon and influx of protumorigenic myeloid cells. Although ETBF has been shown to trigger epigenetic modifications (32, 42, 43), it is unknown whether ETBF induces specific somatic gene mutations capable of driving tumor formation.

Herein, we hypothesized that ETBF colonization increases mutation frequency in CECs, resulting in *Apc* mutations and mutations in other tumor suppressors or proto-oncogenes, which enhance distal colon tumor formation. To test this hypothesis, we utilized two murine models and performed whole-exome and whole-genome sequencing on tumors isolated after ETBF and sham colonization of *Apc*^min/+^ and *Apc*^min/+^*Msh2^fl/fl^*VC mice. Additionally, using methods that previously revealed a specific mutational signature induced by polyketide synthase-positive Escherichia coli (*pks+* E. coli) (44), we cocultured ETBF with human colon organoids to determine if our murine results were replicable in human cells. Our approach was designed to allow us to explore whether ETBF colonization leads to mutations in *Apc* and other tumor suppressors or proto-oncogenes, characterize the genome-wide impact of ETBF colonization on mutation frequency, and analyze the mutational signatures present most frequently in cells exposed to ETBF.

## RESULTS

### ETBF-induced colon tumors undergo *Apc* LOH in *Apc*^min/+^ mice and *Apc* mutagenesis in *Apc*^min/+^*Msh2^fl/fl^*VC mice.

**FIG 1:**
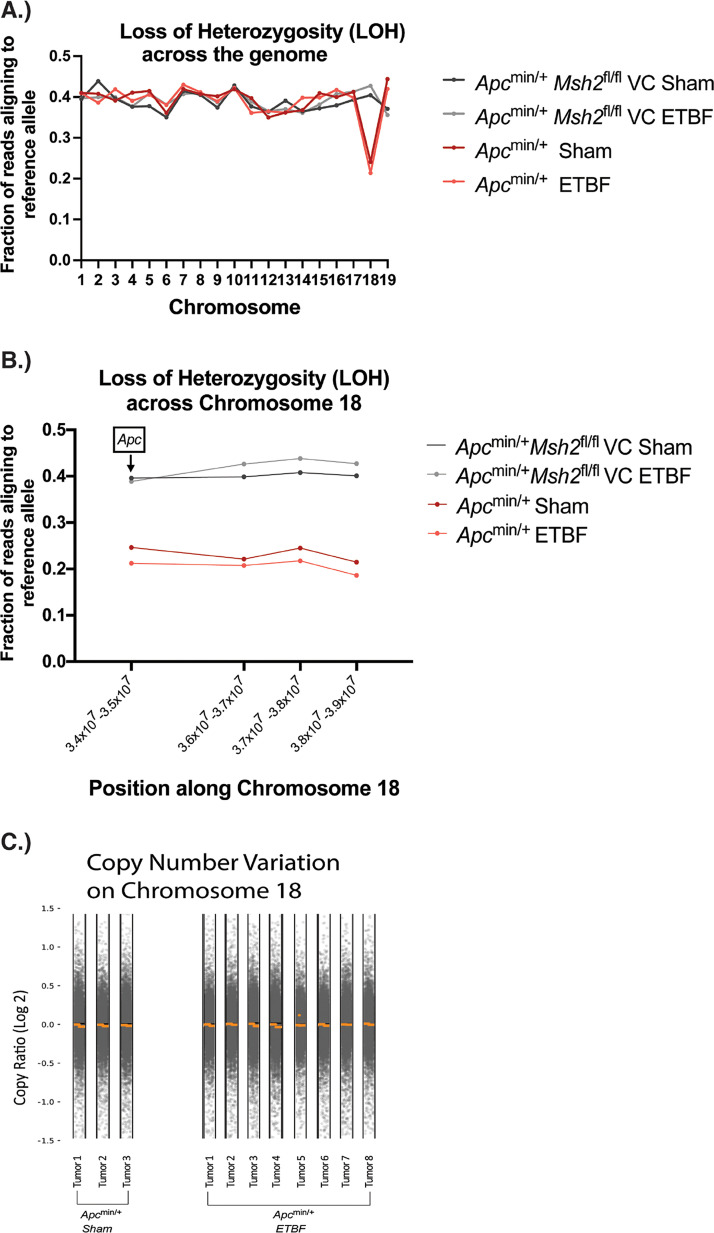
Copy number neutral loss of heterozygosity (LOH) is seen on chromosome 18 in tumors isolated from *Apc*^min/+^ mice. (A) LOH score for each autosomal chromosome. For each chromosome, all positions along the chromosome were averaged to create an LOH score for that chromosome. Only chromosomes with large regions of LOH will show a significantly decreased fraction of reads aligning to the reference allele. (B) Location of LOH along chromosome 18. The position on the *x* axis represents the median chromosomal position taken from the following four intervals along chromosome 18: 3.4 × 10^7^ to 3.5 × 10^7^, 3.6 × 10^7^ to 3.7 × 10^7^, 3.7 × 10^7^ to 3.8 × 10^7^, and 3.8 × 10^7^ to 3.9 × 10^7^. The *Apc* gene is located in the first interval. Error bars represent standard error of the mean. At a position that has lost heterozygosity, the fraction of reads aligning to the reference allele is expected to be considerably lower than 0.5. (C) Copy number variations on chromosome 18 in *Apc*^min/+^ sham and *Apc*^min/+^ ETBF tumor samples as determined by CNVkit. Each gray dot represents an individual data point, and the orange lines/dot represent the average copy number variation over a given region of chromosome 18. A log_2_ copy ratio of 0 represents no difference in copy number at that location between the tumor sample and a normal sample from the same mouse.

Because the majority of tumors which develop in the small intestine of *Apc*^min/+^ mice have undergone *Apc* LOH (45), we first examined all tumor samples (Table 1) for LOH across the genome. In doing this, we only detected an LOH signal on chromosome 18, which contains the *Apc* gene (Fig. 1A). To determine if *Apc* LOH was present, we next looked specifically at a locus within *Apc* on chromosome 18 and 4 megabase pairs downstream of *Apc*; this was the furthest position downstream of *Apc* for which we had consistent data for all groups. All *Apc*^min/+^ sham and *Apc*^min/+^ ETBF tumors showed LOH at the locus within *Apc* and 4 megabase pairs downstream of *Apc* (Fig. 1B; see also Data Set S1 in the supplemental material). In stark contrast, a consistent LOH signal was not identified in any of the *Apc*^min/+^*Msh2^fl/fl^*VC sham or *Apc*^min/+^*Msh2^fl/fl^*VC ETBF tumors (Fig. 1A and B; Data Set S1). When we analyzed copy number variation to determine if the LOH seen in *Apc*^min/+^ mice was accompanied by complete chromosome loss, we saw no significant copy number alterations on chromosome 18 or any other chromosome in either the whole-exome or whole-genome sequencing data (Fig. 1C; see also Fig. S1 in the supplemental material). These results indicate that *Apc*^min/+^ sham and *Apc*^min/+^ ETBF tumors undergo copy number neutral LOH only in chromosome 18 in our murine model.

**TABLE 1:** Mouse tumor samples

Mouse genotype	Mouse no.	Exptl condition	Colon tumor no.^*a*^	No. of tumors analyzed^*b*^
*Apc* ^min/+^	682	sham	1	1
*Apc* ^min/+^	735	sham	2	2
*Apc* ^min/+^	678	ETBF	32	4
*Apc* ^min/+^	710	ETBF	30	4
*Apc*^min/+^*Msh2^fl/fl^*VC	877	sham	1	1
*Apc*^min/+^*Msh2^fl/fl^*VC	966	sham	1	1
*Apc*^min/+^*Msh2^fl/fl^*VC	879	ETBF	17	3
*Apc*^min/+^*Msh2^fl/fl^*VC	965	ETBF	23	3

a Tumors from *Apc*^min/+^ mice were 3 mm in diameter, and tumors from *Apc*^min/+^*Msh2^fl/fl^*VC mice were 1 to 2 mm in diameter.

b Tumors represent those analyzed by exome sequencing. For whole-genome sequencing, the two tumors sequenced from sham-inoculated *Apc*^min/+^*Msh2^fl/fl^*VC mice (mice 877, 966) were pooled, and one tumor sample was sequenced from mice 735, 710, and 965.

We next analyzed our whole-exome sequencing data for additional mutations in *Apc* in the 8 tumors dissected from *Apc*^min/+^*Msh2^fl/fl^*VC mice. A total of 5/6 tumor samples from *Apc*^min/+^*Msh2^fl/fl^*VC ETBF mice possessed mutations in *Apc*, while 0/2 tumor samples from *Apc*^min/+^*Msh2^fl/fl^*VC sham mice possessed mutations in *Apc*. The location and type of mutation seen differed for each tumor as follows: three frameshift mutations (exons 11, 14, 16), one nonsense mutation (exon 14), and one splice site mutation (exon 12). Each of these mutations is predicted to result in a truncated Apc protein. We also performed whole-genome sequencing of the 2 tumor samples isolated from *Apc*^min/+^*Msh2^fl/fl^*VC sham mice in order to identify *Apc* mutations missed by whole-exome sequencing; whole-genome sequencing revealed no additional mutations in *Apc*.

Taken together, our *Apc* gene analysis revealed that in the majority of tumors, either copy number neutral LOH on chromosome 18 (*Apc*^min/+^ mice) or an independent, protein-truncating mutation in *Apc* (*Apc*^min/+^*Msh2^fl/fl^*VC mice) was present, resulting in two gene “hits” in *Apc*.

### ETBF does not significantly enhance overall genome mutation frequency.

Because ETBF induces DNA damage and causes double-stranded DNA breaks (46), we hypothesized that ETBF-induced tumors would contain more single nucleotide variants (SNVs) and indels than sham tumors and that this increase in mutation frequency would impact genes important to CRC development, other than *Apc*. Similarly, because *Msh2* is involved in the recognition and repair of DNA damage, we hypothesized that the increase in mutation frequency with ETBF colonization would be exacerbated in *Apc*^min/+^*Msh2^fl/fl^*VC mice.

**FIG 2:**
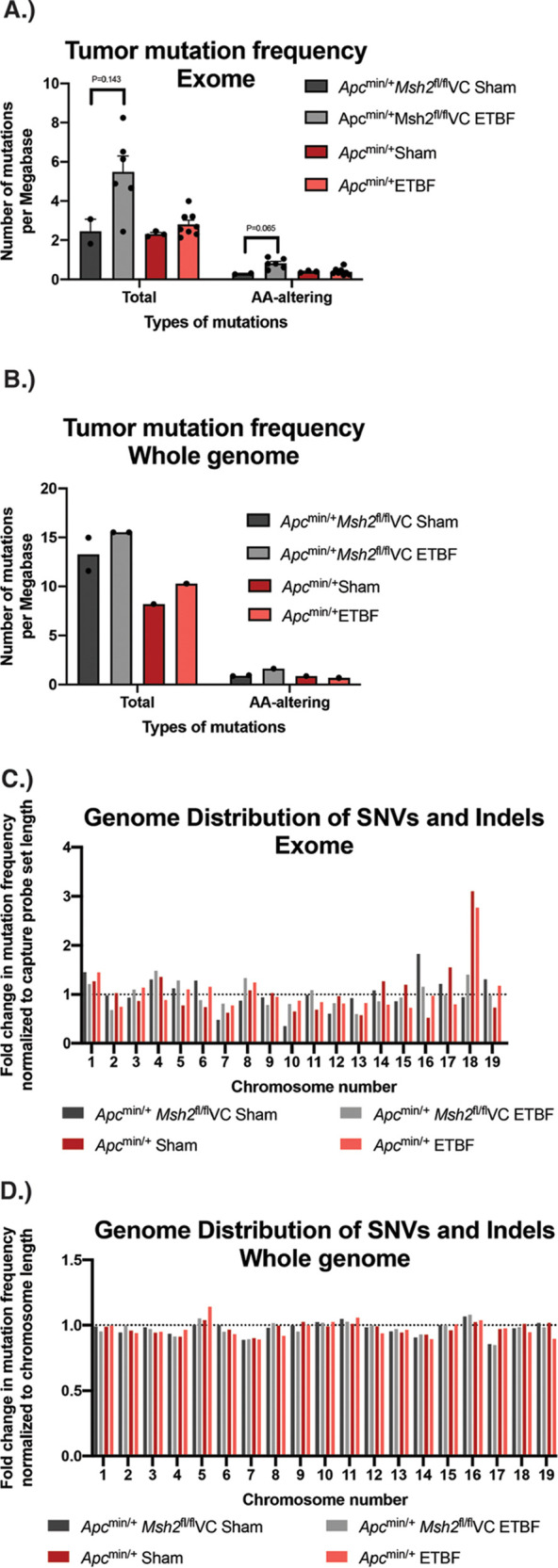
Mutation frequency and distribution in ETBF-induced tumors and sham tumors. (A and B) Number of combined SNVs and indels identified via exome and whole-genome sequencing analyses. The number of mutations present in each group per megabase pair of sequenced DNA is identified. Total number of mutations and mutations resulting only in amino acid changes (amino acid altering) are presented. Error bars represent standard error of the mean. (C and D) Distribution of SNVs and indels by chromosome across the autosomal chromosomes via exome and whole-genome sequencing analyses. For exome sequencing, results were normalized to the overall length of hybrid probes used to target exons on that chromosome. For whole-genome sequencing, results were normalized to the length of each chromosome. The dotted line at 1 indicates the expected value if the mutational burden for each chromosome was evenly distributed. Values greater than 1 represent a higher-than-expected mutation rate for each chromosome, and values less than 1 represent a lower-than-expected mutation rate for each chromosome.

Our exome sequencing data showed that in *Apc*^min/+^ mice, tumors taken from ETBF-colonized mice possessed a mutational frequency similar to sham tumors (2.62 versus 2.29 mutations per megabase when total mutations were analyzed, and 0.24 versus 0.40 mutations per megabase when mutations resulting in amino acid changes [amino acid-altering mutations] were analyzed) (Fig. 2A). In contrast, in *Apc*^min/+^ *Msh2^fl/fl^*VC mice, tumors taken from ETBF-colonized mice showed a trend toward an increased mutational frequency when compared to sham tumors, although it did not reach statistical significance using a Mann-Whitney U test. Specifically, this trend was seen when amino acid-altering mutations were analyzed (*P* = 0.065) with a mutational frequency of 0.79 and 0.30 mutations per megabase for ETBF and sham tumors, respectively. When total mutations were analyzed, the difference in mutational frequency between ETBF and sham tumors doubled (5.5 versus 2.45 mutations per megabase, respectively) but did not approach statistical significance (*P* = 0.143) (Fig. 2A). Qualitative analysis of our whole-genome sequencing data showed similar results (Fig. 2B).

Further, in our exome sequencing data, the number of mutations found on each chromosome roughly aligned with the overall length of hybrid probes used to capture exons on that chromosome for all chromosomes except chromosome 18. Interestingly, only *Apc*^min/+^ sham and *Apc*^min/+^ ETBF tumors showed a similar increased mutation rate on chromosome 18 (Fig. 2C). We think that this isolated increase in mutation rate is likely a consequence of LOH on chromosome 18 in tumors isolated from *Apc*^min/+^ mice for the following reasons: (i) the pattern was not seen in the whole-genome sequencing data (Fig. 2D), (ii) the pattern was not seen in tumors isolated from *Apc*^min/+^*Msh2^fl/fl^*VC mice in which LOH on chromosome 18 was not observed, and (iii) the mutated genes on chromosome 18 are almost exclusively found in a cluster of protocadherin genes and predicted genes expressed primarily in the brain (47) and thus presumably unrelated to CRC.

Additional observations support that an ETBF-induced increase in overall genome mutations is likely not contributory to CRC pathogenesis. First, when the PANTHER classification system was used to identify pathways, biological processes, cellular components, or molecular functions overrepresented in the genes mutated in our tumor samples, no difference between ETBF and sham tumors was seen (data not shown). Second, the only mutated gene identified via whole-exome sequencing in our tumor samples that has been previously linked to CRC as categorized by the Integrative Genomics Viewer (IGV) (48), other than *Apc*, is *Tcf7l2* (49). This gene was mutated in only one of the eight *Apc*^min/+^ ETBF tumors analyzed. Third, no additional mutated tumor suppressors or proto-oncogene (driver genes) were identified in any of the tumor samples via whole-exome sequencing, including the 3 tumors lacking LOH or an *Apc* mutation in *Apc*^min/+^*Msh2^fl/fl^*VC mice. Fourth, while our whole-genome sequencing analysis revealed several mutations in genes associated with CRC in the two tumor samples taken from sham-inoculated *Apc*^min/+^*Msh2^fl/fl^*VC mice (*Mapk9*, *Fos*, *Tgfbr1*), these mutations were all located in regulatory regions, such as the 5′ untranscribed region (UTR), 3′ UTR, and promoter, so their functional importance is difficult to ascertain (see Table S1 in the supplemental material). Taken together, a significant increase in genome mutation frequency in response to ETBF colonization was not detected. However, ETBF colonization may lead to increased genome SNVs and indels in mice with a disruption of the mismatch repair system. In tumors lacking *Apc* LOH or mutations, mutations of other tumor suppressor and proto-oncogenes were not identified.

### Mutation profiles in tumors isolated from sham and ETBF-colonized mice are highly similar and both implicate dysfunctional DNA damage responses in tumor formation.

We next analyzed the mutational profiles of both sham and ETBF-induced tumors to determine if colonization with ETBF produced a specific mutational signature as previously reported for *pks+* E. coli (44). In accordance with the mutation categories and subcategories defined in Alexandrov et al. (50), we created indel mutational profiles in the 83-mutation type format and SNV mutational profiles in the 6-mutation type and 96-mutation type formats. Categories of indels and SNV mutations were categorized as described in Materials and Methods (mutational profiles analysis).

**FIG 3:**
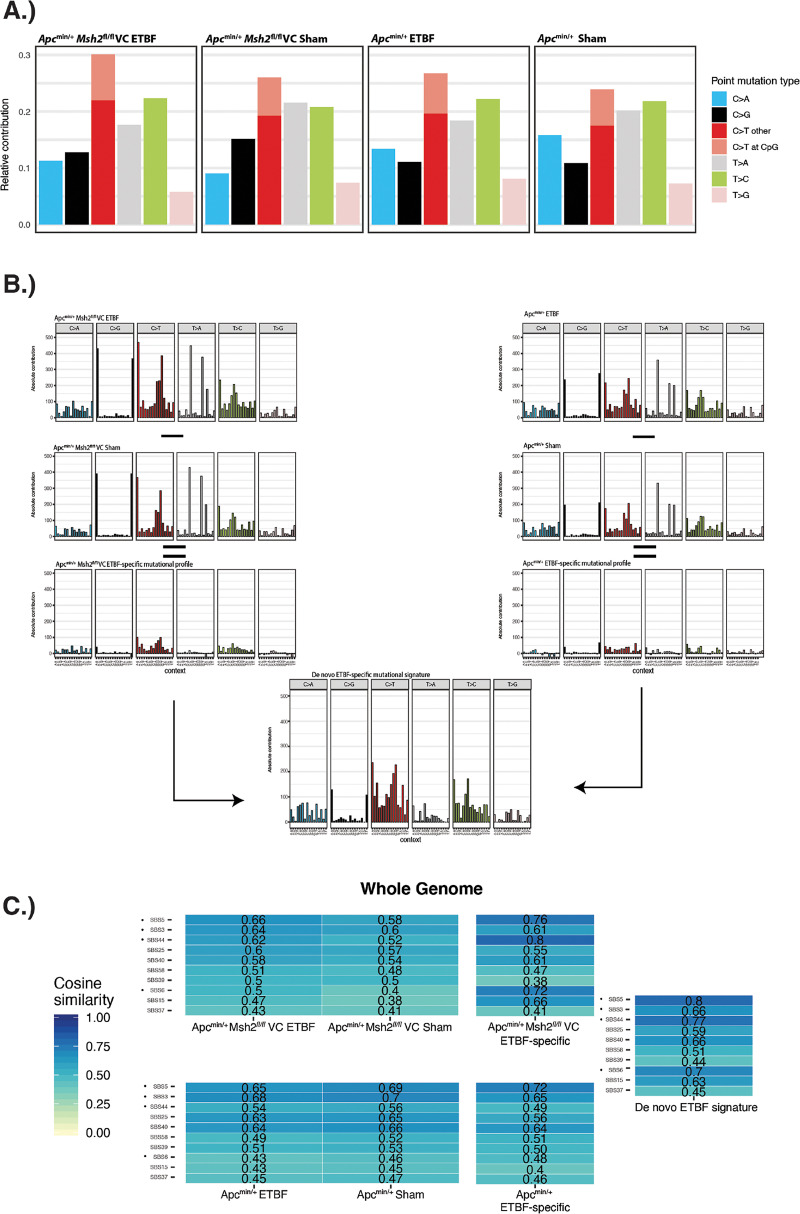
ETBF-specific mutational profiles extracted from whole-genome sequencing and compared to COSMIC single-base substitution (SBS) signatures. The R package MutationalPatterns was used to create SBS mutational profiles. (A) SNV mutational profile in the 6-mutation type format for whole-genome sequencing data. In the 6-mutation type format, mutations are divided into the following 6 categories: C > A, C > G, C > T, T > A, T > C, and T > G. Additionally, C > T mutations are further subdivided into those that occur within a CpG dinucleotide context and those that do not. (B) Graphic detailing how the ETBF-specific mutational profiles and the *de novo* extracted ETBF-specific mutational signature were created from the whole-genome sequencing data in the 96-mutation type format. In the 96-mutation type format, the 6 mutations outlined above are further subdivided into 16 categories, which represent the 16 combinations of nucleotides immediately 5′ and 3′ to each mutated base. The *de novo* signature was extracted from the ETBF-specific mutational profiles in *Apc*^min/+^ and *Apc*^min/+^*Msh2^fl/f^* VC mice. The total number of mutations belonging to each trinucleotide mutation type is presented. (C) Heatmaps comparing SBS COSMIC signatures (vertical axis) to the mutational profiles created from whole-genome sequencing data in *Apc*^min/+^ mice and *Apc*^min/+^*Msh2^fl/fl^*VC mice. Numbers displayed represent “cosine similarity,” which is a metric used to quantify the similarity between any two mutational matrices. Only the top 10 COSMIC SBS signatures are shown. Dots indicate mutational profiles most similar to ETBF signature across multiple analyses.

Our indel analysis did not show any differences between sham and ETBF tumors in either mouse strain (see Fig. S2A and B in the supplemental material). In all samples, the mutational profiles were dominated by two general categories as follows: single-base insertions/deletions at sites of single-base repeats numbering greater than 6 units in length or two-base deletions at sites of two-base repeats numbering greater than 6 units in length. The former represents COSMIC signatures associated with slippage during DNA replication (termed ID1 and ID2) and is found in the majority of human tumor samples (51). The latter most closely resembles ID12, which has no known etiology. The SNV 6-mutation type format showed few differences between the 4 tumor groups. All groups were dominated by C > T mutations, and all groups possessed a majority of C > T mutations that occurred outside of the CpG dinucleotide context in both the whole-genome and exome analyses (Fig. 3A; see also Fig. S3A in the supplemental material). Analysis of the SNV 96-mutation type format also revealed similar profiles between groups, though differences were more apparent in the exome sequencing data (Fig. S3B) than the whole-genome sequencing data (Fig. 3B).

To pinpoint small but potentially relevant differences, we generated ETBF-specific mutational profiles by subtracting the sham tumor mutational profile from the ETBF tumor mutational profile separately in both *Apc*^min/+^ mice and *Apc*^min/+^*Msh2^fl/fl^*VC mice. This method allowed us to eliminate the background mutational profile present in each mouse genotype and specifically identify those mutations that are unique to ETBF-induced tumors (Fig. 3B; see also Fig. S3B). This technique was recently used by Pleguezuelos-Manzano et al. to elucidate the mutational signature associated with *pks+* E. coli (44). To identify a pattern of mutations common to ETBF tumors in both *Apc*^min/+^ and *Apc*^min/+^*Msh2^fl/fl^*VC mice, we used nonnegative matrix factorization within the MutationalPatterns R package to extract a combined *de novo* ETBF-specific mutational signature from the ETBF-specific mutational profiles from each mouse genotype (Fig. 3B; see also Fig. S3B). Though the *de novo* ETBF-specific mutational profile was derived from both mouse strains, we note that it was most similar to tumors taken from *Apc*^min/+^*Msh2^fl/fl^*VC mice (cosine similarity of 0.98 versus 0.83). Finally, we compared all of our mutational profiles to published human single-base substitution (SBS) mutational signatures extracted from the COSMIC database (51). Our results from these analyses are presented in Fig. 3C and Fig. S3C (whole-genome and exome sequencing data, respectively) and discussed below.

Because whole-genome sequencing allows for the detection of more mutations than exome sequencing, we used this approach in initial samples as the mutational profiles created from that data can be more reliably compared to the COSMIC SBS signatures. Secondarily, we used an increased sample number analyzed by exome sequencing to validate the whole-genome results, seeking to ensure that conclusions drawn are not due to sampling error. Using this approach, our whole-genome sequencing data in *Apc*^min/+^ mice showed SBS5 and SBS3 with the highest degree of similarity to the mutational profiles generated from sham tumors and ETBF-induced tumors, as well as in the ETBF-specific mutational profile (Fig. 3C; see also Fig. S4A in the supplemental material). The etiology of SBS5 is unknown but proposed to be a clock-like signature, and SBS3 is associated with defective homologous recombination-based DNA repair (51, 52). In *Apc*^min/+^*Msh2^fl/fl^*VC mice, SBS5 and SBS3 again showed the highest degree of similarity to the mutational profiles generated from sham tumors and ETBF-induced tumors, but SBS44 showed the highest degree of similarity to the ETBF-specific mutational profile, followed by SBS5 and then SBS6 (Fig. 3C; Fig. S4A). Both SBS44 and SBS6 are associated with defective DNA mismatch repair. The *de novo* ETBF-specific mutational profile derived from both mouse genotypes showed the highest degree of similarity to SBS5, SBS44, SBS6, and then SBS3 (Fig. 3C; Fig. S4A). Analysis of our exome sequencing data showed similar results: SBS5, SBS44, SBS6, and SBS3 were among the top 10 COSMIC SBS signatures most similar to the sham, ETBF, ETBF-specific, and the *de novo* ETBF-specific mutational profiles (Fig. S3C and S4B).

Taken together, our mutational profile analysis suggests that ETBF-induced tumors arise via the same mechanisms as spontaneous (sham) tumors in our mouse model, which include defective DNA-mismatch repair in *Apc*^min/+^*Msh2^fl/fl^*VC mice and defective homologous recombination-based repair in *Apc*^min/+^ mice. Additionally, other mechanisms likely contribute to ETBF-induced tumor formation that have yet to be fully understood; namely, SBS5 was featured prominently in our analyses and is known to accumulate linearly with age in several cancers, but its exact etiology is unknown (52).

### ETBF-induced mutational analyses are replicated in an organoid model of ETBF colonization.

Pleguezuelos-Manzano et al. used an organoid-bacteria coculture model to better understand the impact of gut bacterial colonization on colon epithelial cells (44). Here, we used a similar approach by microinjecting ETBF or a DYE control into the lumen of human colon organoids. The organoids were passaged and reinjected with bacteria several times before individual cells were cloned and analyzed via whole-genome sequencing. We first determined the mutational frequency in organoids exposed to ETBF and DYE control. We then used the same approach described previously to examine indel mutation profiles in the 83-mutation type format and SNV mutational profiles in the 6-mutation type format and the 96-mutation type format. Finally, we compared those profiles to the published COSMIC SBS signatures.

**FIG 4:**
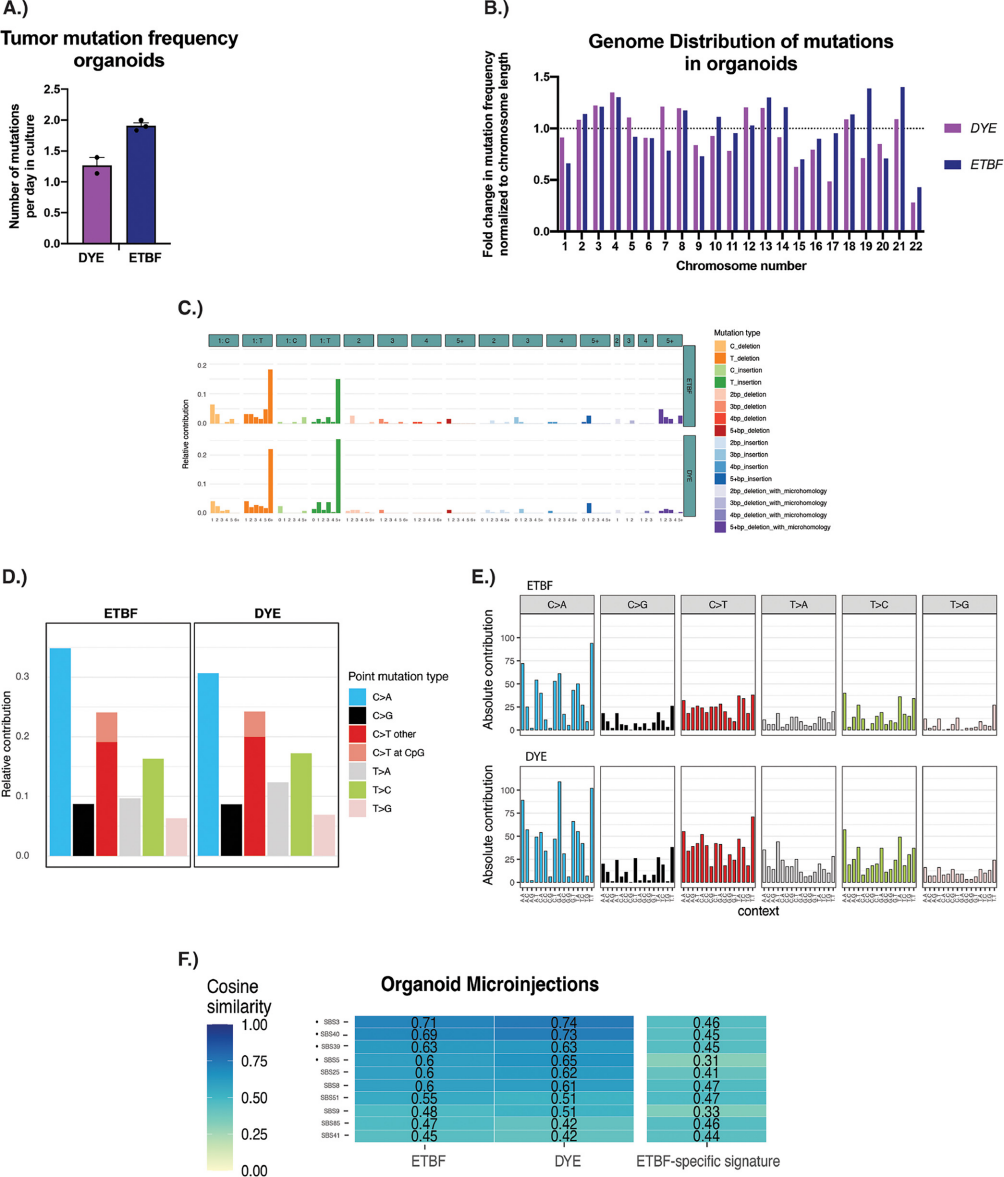
ETBF-induced mutational analyses in an organoid model of ETBF-colonization. (A) Plot showing the number of combined SNVs and indels identified via whole-genome sequencing analysis. The mutation rate per day in culture is identified. Error bars represent standard error of the mean. To determine the mutational load estimate during culture, mutation numbers were divided by the number of days the samples have been in culture. (B) Distribution of mutations throughout the genome. Only autosomal chromosomes are shown. Results were normalized to the length of each chromosome. The dotted line at 1 indicates the expected value if the mutational burden for each chromosome was evenly distributed. (C) Indel mutational profiles in the 83-mutation type format. This format groups indels based on several criteria including size of the indel, nucleotides affected, and the presence of the indel in a repetitive region and/or microhomology region. (D) SNV mutational profile in the 6-mutation type format. In the 6-mutation type format, mutations are divided into 6 categories as follows: C > A, C > G, C > T, T > A, T > C, and T > G. Additionally, C > T mutations are further subdivided into those that occur within a CpG dinucleotide context and those that do not. (E) SNV mutational profile in the 96-mutation type format. In the 96-mutation type format, the 6 mutations outlined above are further subdivided into 16 categories, which represent the 16 combinations of nucleotides immediately 5′ and 3′ to each mutated base. (F) Heatmaps comparing SBS COSMIC signatures (vertical axis) to the mutational profiles created from whole-genome sequencing data in organoids. Numbers displayed represent “cosine similarity,” which is a metric used to quantify the similarity between any two mutational matrices. Only the top 10 COSMIC SBS signatures are shown. Dots indicate mutational profiles most similar to ETBF signature.

The number of mutations detected in organoids cocultured with ETBF was comparable to the number of mutations detected in control (DYE) organoids (1.90 versus 1.27 mutations per day in culture; *P* = 0.20) (Fig. 4A). Similar to the mouse model results, the mutations were distributed throughout the genome in the organoid model (Fig. 4B). Some variation was seen between chromosomes, but this is to be expected, as the overall number of mutations detected in the organoid model was relatively low when compared to our mouse model (Fig. 4A versus Fig. 2A and B). The ID mutational signatures showed few differences between DYE control and ETBF cocultured organoids and possessed a cosine similarity of 0.93. Both profiles were most similar to signatures ID1 and ID2 (Fig. 4C), the same signatures featured prominently in our mouse model. The 6-mutation type analysis also showed very few differences between ETBF and DYE control organoid mutational profiles. C > A mutations were present most frequently, followed by C > T mutations (Fig. 4D). This differs slightly from what was seen in our mouse model, where C > A mutations were among the least frequent mutation type, but is not unexpected as mutation accumulation *in vitro* has been shown to be associated with oxidative stress and an increased frequency of C > A mutations (53).

Analysis of the 96-mutation type format also revealed similar mutational profiles between the ETBF and DYE control group, with a cosine similarity of 0.955 (Fig. 4E). When these mutational profiles were compared to the COSMIC SBS mutational signatures, SBS3 emerged as the signature most similar to both the DYE control and ETBF mutational profiles. SBS5 was also among the top 10 most similar signatures, along with SBS39 and SBS40, both of which have an unknown etiology (Fig. 4F; see also Fig. S4C). SBS44 and SBS6 were not among the most similar COSMIC SBS mutational signatures, which is consistent with the fact that the human organoids did not have a disrupted mismatch repair system. Consistent with our murine model, these results suggest that in an organoid coculture model, ETBF does not induce mutations and does not result in a unique mutational profile.

## DISCUSSION

Previous studies have shown that ETBF can lead to changes in the genome (DNA damage) and epigenome (DNA methylation, chromatin accessibility changes) (32, 42, 43, 54, 55), but the specific mechanisms by which ETBF modifies DNA to induce tumor formation has not been established. Herein, we have attempted to bridge this gap by performing whole-exome and whole-genome sequencing on tumors isolated from sham and ETBF-colonized *Apc*^min/+^ and *Apc*^min/+^*Msh2^fl/fl^*VC mice and whole-genome sequencing of organoids cocultured with ETBF.

Our results indicate that ETBF-induced tumors in the distal colon of *Apc*^min/+^ mice form via *Apc* LOH, the same mechanism as spontaneous (sham) tumors in our mouse model, various other *Apc* mutant mouse models (27, 45), and approximately 50% of sporadic colorectal tumors (25, 56, 57). In *Apc*^min/+^ mice, tumors do not have ETBF-induced mutations in *Apc* nor do they have additional mutations in other tumor suppressors or proto-oncogenes. Thus, these data suggest that ETBF-induced tumors are nearly identical genetically to sham tumors, and both form via copy number neutral LOH when a first “hit” in *Apc* exists.

Our primary conclusion is corroborated by human colon organoid experiments showing that the mutational profiles of organoids (comprised only of colon epithelial cells, the CRC target cell) exposed to ETBF are nearly identical to the profile of organoids not exposed to ETBF. *In vivo* and *in vitro*, these profiles are most similar to COSMIC SBS signatures associated with errors in homologous recombination, DNA damage repair, and clock-like mutagenesis mechanisms. These results differ drastically from those reported for *pks+* E. coli in which a recent study (44) identified a bacteria-induced pattern of DNA mutations in CECs via direct exposure of normal human colonoids *in vitro* to *pks+* E. coli. In contrast, we show herein that ETBF does not induce a unique pattern of mutation.

Nonetheless, ETBF markedly increases and accelerates colon tumorigenesis in both *Apc*^min/+^ and *Apc*^min/+^*Msh2^fl/fl^*VC mice (Table 1) (33, 35). ETBF-induced Th17-predominant myeloid inflammatory cell infiltration and induction of CEC proliferation, both necessary for tumor formation, likely contribute to the impact of ETBF in colonized individuals (33, 35). In particular, we hypothesize that in a permissive host (i.e., in an individual with subclinical inflammation or a preexisting first “hit” in *APC*), ETBF transitions from relatively harmless colonizer to procarcinogenic bacterium. This is supported by prior data, which show that FAP patients at the time of colectomy display mucus-invasive colon mucosal biofilms comprised predominantly of *pks+* E. coli and ETBF throughout their colons (23). Additionally, prior reports have shown that even in FAP families with individuals displaying the same *APC* mutation that the pace of colon tumorigenesis is heterogenous (58). We propose that ETBF, and likely other microbes, contributes to this heterogeneity in hereditary CRC. Importantly, our current data do not support a role for increased ETBF-induced mutagenesis leading to tumor formation in these patients. Instead, other mechanisms, including inflammation-induced (e.g., IL-17, reactive oxygen species) and/or epigenetic modifications, likely modify colon epithelial cell context and tumor potential in a permissive host.

In contrast, ETBF-induced tumors in the distal colon of *Apc*^min/+^ *Msh2^fl/fl^*VC mice most often form via a protein-truncating mutation in the *Apc* gene, the same mechanism as spontaneous (sham) tumors in multiple mouse models with both an *Apc* mutation and a mutation in the mismatch repair system (59, 60). In mice with a dysfunctional mismatch repair system, ETBF increases the odds of any given colon epithelial cell receiving a second protein-truncating mutation in *Apc*. The distribution of mutations throughout the genome in ETBF-induced tumors (Fig. 2C and D; Fig. 4B) suggests that ETBF does not target *Apc*; instead, mutations accumulate everywhere, but only mutations in *Apc* are advantageous, and thus selected for, when a first “hit” in *Apc* exists.

Early studies have shown that Bacteroides fragilis is enriched in individuals with Lynch syndrome (61), but whether this enrichment represents ETBF strains specifically has yet to be investigated. If so, the possible increased mutation frequency induced by ETBF (Fig. 2A), the timing of acquisition of ETBF colonization, and the host mucosal immune response to ETBF may help explain the extreme variability in rate of tumor formation seen in individuals with Lynch syndrome, a poorly understood phenomenon (62).

The microbiome field has long sought to answer the question of whether any microbe and/or microbial community is sufficient to initiate CRC. *pks+* E. coli has recently been associated with a specific mutational signature, strengthening its claim as a CRC initiator (44). Herein, our data suggest that ETBF does not produce a specific mutational signature. Instead, tumors in ETBF-colonized mice develop via pathways typically dominant in the specific genetic background examined: for cells with a functional mismatch repair system, that is *Apc* LOH, and for cells with a dysfunctional mismatch repair system, that is via increased point mutations and small insertions/deletions. Thus, available data suggest that *pks+* E. coli may initiate CRC, whereas ETBF may promote CRC through predominantly nongenomic mechanisms.

As a next step, the specific genetic changes present in human colon tumors from individuals colonized with potentially carcinogenic bacteria should be analyzed. Novel ETBF-induced genomic mutations or a significant increase in genome mutation rate were not identified in our study, potentially due to our relatively small sample size. As the cost of sequencing decreases, efforts should be made to sequence larger numbers of samples with and without exposure to ETBF to definitively determine if exposure to ETBF alters mutation frequency in normal CECs. Additionally, it is imperative that these types of analyses be extended to other bacteria linked to CRC, such as *Fusobacterium* spp. and polymicrobial biofilms, in order to better identify individuals at increased risk of developing CRC due to their gut microbiome compositions.

## MATERIALS AND METHODS

### Mouse inoculations.

Min*^ApcΔ^*^716+/−^ mice (referred to as *Apc*^min/+^ mice) express a mutant *Apc* that contains a truncation at amino acid 716. *Apc*^min/+^*Msh2^fl/fl^*VC mice possess the aforementioned *Apc* mutation and an additional mutation in *Msh2*. In these mice, the *Msh2* gene is flanked by LoxP sequences, and Cre expression is driven by the *villin* promoter, which causes deletion of *Msh2* only in intestinal epithelial cells (*Msh2^fl/fl^*VC mice). *Apc*^min/+^ mice and *Apc*^min/+^ Msh2*^fl/fl^*VC mice were housed at two different facilities (*Apc*^min/+^ mice at Johns Hopkins School of Medicine and *Apc*^min/+^ Msh2*^fl/fl^*VC mice at Indiana University School of Medicine). All mouse strains used were specific pathogen free. Clindamycin (0.1 g L^−1^) and streptomycin (5 g L^−1^) were administered 3 to 5 days before ETBF or sham inoculation in order to enhance stable ETBF colonization (33). ETBF strain 86-5443-2-2 (a clindamycin-resistant isolate from piglet; kindly provided by Lyle Myers, Montana State University) was used; all B. fragilis strains are aminoglycoside resistant (36). For the ETBF inoculation, ~1 × 10^8^ bacteria were administered in phosphate-buffered saline (PBS) via oral gavage, and for the sham inoculation, PBS was administered alone via oral gavage. Inoculations were performed when mice were approximately 4 weeks old. For *Apc^min/+^* mice, bacterial colonization was confirmed by stool culture at 1 week and 3 months postinoculation (~5 × 10^10^ and ~4 × 10^10^ CFU/gm stool, respectively). All mouse protocols were approved by the Johns Hopkins University and Indiana University School of Medicine Animal Care and Use Committees in accordance with the Association for Assessment and Accreditation of Laboratory Animal Care International.

### Organoid culture.

The clonal culture of the human intestinal organoid line ASC-5a, which was used in this study, was described previously (63). Organoids were cultured in droplets of Cultrex PathClear reduced growth factor basement membrane extract (BME) (R&D Systems/Bio-Techne; catalog no. 3533-001) covered by medium containing Advanced DMEM/F12 (Gibco), 1× B27, 1× GlutaMAX, 10 mmol/L HEPES, 100 U/mL penicillin-streptomycin (all Thermo-Fisher), 1.25 mM *N*-acetylcysteine, 10 μM nicotinamide, 10 μM p38 inhibitor SB202190 (all Sigma-Aldrich), and the following growth factors: 0.5 nM WNT surrogate-Fc fusion protein, 2% noggin conditioned medium (both U-Protein Express), 20% Rspo1 conditioned medium (in-house), 50 ng/mL epidermal growth factor (EGF) (Peprotech), 0.5 μM A83-01, and 1 μM PGE2 (both Tocris). For the generation of subclonal cultures after bacterial exposure, organoids were dissociated to single cells and seeded at a density of 50 cells/μL. At this step, the culture medium was supplemented with ROCK inhibitor Y-27632 (10 μM; AbMole; M1817) during the first outgrowth week. Single cell-derived organoids were then individually picked and transferred to an independent well.

### Organoid-bacteria coculture.

Organoids were cultured in antibiotics-free medium at least 2 days prior to the coculture experiment. The ETBF strain 86-5443-2-2, referenced previously (36), was inoculated in brain heart infusion broth supplemented with 5 g/L yeast extract (BD Bacto), 0.5 g/L l-cysteine (Sigma), 10 mL/L Hemin solution (Sigma), 0.2 mL/L vitamin K1 (Sigma), and 6 μg/mL clindamycin (Sigma) and incubated in an anaerobic chamber overnight. Bacteria were reinoculated on the day of injection, grown to an optical density of 0.8 and washed once with advanced DMEM (Gibco) supplemented with GlutaMAX and HEPES. Then, bacteria were injected at a multiplicity of infection of 1 together with 0.05% (wt/vol) Fast Green dye (Sigma). At this point, 5 μg/mL of the nonpermeant antibiotic gentamicin was added to the medium to prevent bacteria overgrowth outside the organoid lumen. ETBF was killed with 1× Primocin (InvivoGen) after 3 days of coculture, after which organoids were left to recover for 4 days before being passaged. When the organoids reached a cystic stage again (typically after 2 to 3 weeks), the injection cycle was repeated. This procedure was repeated three times for ETBF-exposed organoids. To compare the additive mutagenic effects of ETBF, exposure of control conditions was performed. Organoid clones derived from cultures exposed to Fast Green dye only were sequenced after being exposed over one or eight injection rounds.

### Tumor dissection and DNA extraction.

Two months after ETBF/sham inoculation (for *Apc*^min/+^*Msh2^fl/fl^*VC mice) or 3 months after ETBF/sham inoculation (for *Apc*^min/+^ mice), mice were sacrificed and dissected and the distal colon removed. *Apc*^min/+^*Msh2^fl/fl^*VC mice were sacrificed earlier than *Apc*^min/+^ mice due to poor health. Tumors were grossly dissected from mouse colons, flash frozen using liquid nitrogen, and stored at −80°C. For *Apc*^min/+^*Msh2^fl/fl^*VC mice, these tumors were approximately 1 to 2 mm in diameter and, for *Apc*^min/+^ mice, 3 mm in diameter. Table 1 displays each mouse analyzed and their respective tumor burdens. Of note, gross (i.e., visible) tumors (macroadenomas) were dissected from each mouse distal colon and whole-tumor DNA used for sequencing. Therefore, the DNA sequenced was a mixture of tumor and normal cells. From each mouse, a piece of tissue near the mid-distal colon with no visible tumor was also dissected, flash frozen, and stored at −80°C. From each mouse, the heart was also dissected, flash frozen in liquid nitrogen, and stored at −80°C. For exome sequencing, DNA was extracted from all tissue samples using the MasterPure complete DNA and RNA purification kit (Lucigen). For whole-exome sequencing analysis, at least 500 ng of DNA was used for library preparation and sequencing by the Genetic Resources Core Facility at Johns Hopkins School of Medicine. For whole-genome sequencing analysis, at least 200 ng genomic DNA was used for library preparation and sequencing by Novogene (Sacramento, CA).

### Tumor/normal analysis.

For all analyses, whole-exome sequencing data samples were pooled to create the following 4 groups: *Apc*^min/+^ sham, *Apc*^min/+^ mice which received a sham inoculation; *Apc*^min/+^ ETBF, *Apc*^min/+^mice which received an ETBF inoculation; *Apc*^min/+^*Msh2^fl/fl^*VC sham, *Apc*^min/+^*Msh2^fl/fl^*VC mice which received a sham inoculation; and *Apc*^min/+^*Msh2^fl/fl^*VC ETBF, *Apc*^min/+^*Msh2^fl/fl^*VC mice which received an ETBF inoculation.

### Whole-exome sequencing library preparation.

DNA fragmentation was performed on 50 to 200 ng of genomic DNA using a Covaris E210 system. Exon capture was done using Twist Biosciences double-stranded DNA probes system. The DNA capture was then PCR amplified. The Agilent Bioanalyzer was used for quality control of adequate fragment sizing and quantity of DNA capture. DNA sequencing was performed on an Illumina NovaSeq 6000 instrument using standard protocols for a 100-bp paired-end run. Samples were pooled according to capture size to achieve >90% completeness at a minimum of 20× coverage.

### Whole-genome sequencing library preparation.

For mouse samples, a total amount of 1.0 μg DNA per sample was used as input material for the DNA sample preparations. Sequencing libraries were generated using NEBNext DNA library prep kit following manufacturer's recommendations, and indices were added to each sample. The genomic DNA was randomly fragmented to a size of 350 bp by shearing, then DNA fragments were end polished, A-tailed, and ligated with the NEBNext adapter for Illumina sequencing, and further PCR-enriched by P5 and indexed P7 oligonucleotides. The PCR products were purified (AMPure XP system), and the resulting libraries were analyzed for size distribution by Agilent 2100 Bioanalyzer and quantified using real-time PCR. For organoid samples, genomic DNA was isolated from frozen organoid pellets using a Qiagen DNeasy blood and tissue kit and eluted in 50 μL low EDTA (10 mM Tris base, 0.1 mM EDTA). Sequencing libraries were prepared with 50 ng DNA using a TruSeq Nano kit (Illumina). Libraries were sequenced on a NovaSeq 6000 or HiSeq X10 sequencer at 15× or 30× base coverage for each clone at the Hartwig Medical foundation (www.hartwigsequencingservices.nl).

### Whole-exome sequencing analysis.

Whole-exome sequencing analysis was performed by the Genetic Resources Core Facility at Johns Hopkins School of Medicine. Burrows-Wheeler Aligner (BWA) was utilized to map the paired-end reads to the mm10 reference genome. SAMtools was used to sort and index the original BAM file. Picard was used to mark duplicate reads.

### Whole-genome sequencing analysis.

For mouse samples, whole-genome sequencing analysis was performed by Novogene (Sacramento, CA). Burrows-Wheeler Aligner (BWA) was utilized to map the paired-end clean reads to the mm10 reference genome. SAMtools was used to sort and index the original BAM file. Picard was used to mark duplicate reads. For organoid samples, the previously sequenced clonal parental line was sequenced at 30× using an Illumina HiSeq X10 sequencing machine (44). Sequencing reads from all samples were mapped to the human reference GRCh38 genome using the Burrows-Wheeler Aligner v0.7.5a “BWA-MEM -c 100 -M”. Duplicate sequencing reads were marked using Sambamba v0.6.8. A full description and source code for the NF-IAP version 1.2 pipeline can be retrieved (https://github.com/UMCUGenetics/NF-IAP).

### Variant calling for organoid whole-genome sequencing.

Variants in all samples were called using GATK HaplotypeCaller version 4.1.3.0 using default settings. Variants were filtered using GATK 4.1.3.0 using the following filter settings for SBS: –filter-expression 'QD < 2.0′ –filter-expression 'MQ < 40.0′ –filter-expression 'FS > 60.0′ –filter-expression 'HaplotypeScore > 13.0′ –filter-expression 'MQRankSum < -12.5′ –filter-expression 'ReadPosRankSum < -8.0′ –filter-expression 'MQ0 >= 4 && ((MQ0/(1.0 * DP)) > 0.1)' –filter-expression 'DP < 5′ –filter-expression 'QUAL < 30' –filter-expression 'QUAL >= 30.0 && QUAL < 50.0′ –filter-expression 'SOR > 4.0′ –filter-name 'SNP_LowQualityDepth' –filter-name 'SNP_MappingQuality' –filter-name 'SNP_StrandBias' –filter-name 'SNP_HaplotypeScoreHigh' –filter-name 'SNP_MQRankSumLow' –filter-name 'SNP_ReadPosRankSumLow' –filter-name 'SNP_HardToValidate' –filter-name 'SNP_LowCoverage' –filter-name 'SNP_VeryLowQual' –filter-name 'SNP_LowQual' –filter-name 'SNP_SOR' -cluster 3 -window 10”. The following settings were used to filter all other variants: filter_criteria = “–filter-expression 'QD < 2.0′ –filter-expression 'ReadPosRankSum < -20.0′ –filter-expression 'FS > 200.0′ –filter-name 'INDEL_LowQualityDepth' –filter-name 'INDEL_ReadPosRankSumLow' –filter-name 'INDEL_StrandBias'.”

To obtain high-quality sequencing variant data, we filtered variants using an in-house filtering pipeline SMuRF version 2.1.1 (https://github.com/ToolsVanBox/SMuRF). In short, for every variant, the variant allele frequency (VAF) was determined using a pileup of all mapped reads at the position of the mutation. Variants detected in clonal organoid cultures sequenced at 30× depth were filtered for the following criteria: VAF ≥ 0.3, base coverage ≥ 10, and an MQ quality ≥ 60. In organoid clones sequenced at 15× depth, the following filter settings were altered: VAF ≥ 0.15 and a base coverage of ≥5. In addition, to select only mutations occurring during *in vitro* culture, variants present in the clonal parental line were removed. To remove recurrent mapping or sequencing artifacts, samples were filtered against a blacklist containing sequencing data from healthy bone marrow mesenchymal stromal cells (64).

### Variant calling for *Apc* LOH analysis.

Illumina NovaSeq reads were processed through Illumina’s real-time analysis (RTA) software generating base calls and corresponding base call quality scores. Resulting data was aligned to a reference genome with the Burrows-Wheeler Alignment (BWA) tool resulting in a SAM/BAM file. Postprocessing of the aligned data included local realignment around indels, base call quality score recalibration performed by the Genome Analysis Tool Kit (GATK), and flagging of molecular/optical duplicates using software from the Picard program suite. Per sample variant calling was performed by GATK3.7 according to best practices. Per sample data quality metrics included, but were not limited to, transition/transversion ratios (ts/tv), percent in the database of single nucleotide polymorphisms (dbSNP), capture specificity, and percent of targeted bases covered ≥20×.

### *Apc* LOH analysis.

For analysis of loss of heterozygosity (LOH) in our tumor samples, variant call format (VCF) files produced by HaplotypeCaller from GATK3.7 were used. Normalization was carried out by calculating the absolute difference between the fraction of reads aligning to the reference allele and 0.5 (the expected fraction of reads aligning to the reference allele for a heterozygous sample). This normalization resulted in a value that was always less than or equal to 0.5 for all positions. We identified heterozygous positions as positions for which the fraction of reads aligning to the reference allele was between 0.5 and 0.4. All heterozygous positions in the heart tissue samples (designated as normal) were identified for each mouse. The normal sample for mouse 965 did not pass library prep standards, so the normal sample for mouse 966 (a littermate of mouse 965) was used instead. The positions were filtered for variants with a depth of coverage of at least 20×. We then queried those positions for the fraction of reads aligning to the reference allele in the tumor samples taken from the same mouse. Again, these positions were filtered for variants with a depth of coverage of at least 20× in the tumor samples. Samples were then grouped by inoculation status and mouse genotype, and the average fraction of reads aligning to the reference allele at every position was calculated. For the analysis that examined LOH across the genome, only positions with information from at least 2 of the 4 inoculation groups were included, and zeroes were excluded unless they were supported by more than one data point. For LOH on chromosome 18, the average LOH value was calculated for 4 regular intervals along chromosome 18 as follows: 3.4 × 10^7^ to 3.5 × 10^7^, 3.6 × 10^7^ to 3.7 × 10^7^, 3.7 × 10^7^ to 3.8 × 10^7^, and 3.8 × 10^7^ to 3.9 × 10^7^. All available positions were used.

### Mouse SNV/indel analysis.

For both single nucleotide variant (SNV) and insertion/deletion (indel) analysis, CRAM/BAM files were used by Strelka to identify SNVs and indels. For this analysis, DNA isolated from heart tissue samples was input as “normal,” and DNA isolated from tumor samples extracted from the same mouse was input as “tumor.” Samples were filtered using Strelka’s baseline filtering algorithm and were also filtered at a read depth of 10×. The R package “VariantAnnotation” (65) was then used to annotate each variant. To quantify the genomic distribution of mutations in each sample, the number of mutations identified was normalized to either the length of the exon probes used for each chromosome (whole-exome sequencing analysis) or the length of each chromosome (whole-genome sequencing analysis).

### Copy number variant analysis.

CNVkit (66) uses read-depth to identify duplications and deletions. BAM or CRAM files, aligned to the mm10 reference genome using BWA, were used as input. Copy number variants were identified by comparing tumor samples to heart tissue samples extracted from the same mouse, with the exception of mouse 965. The normal sample for mouse 965 did not pass library prep standards, so the normal sample for mouse 966 (a littermate of mouse 965) was used instead. For CNVkit analysis of the whole-exome sequencing data, the bed file containing the specific target regions used in library preparation was input using the “target” flag. For CNVkit analysis of the whole-genome sequencing data, no target flag was used.

### Mouse mutational profiles analysis.

For mouse tumors, to analyze small indels, the R package SigProfilerMatrixGenerator (https://github.com/AlexandrovLab/SigProfilerMatrixGenerator) (67) version 1.0 was used, and to analyze SNVs, the R package MutationalPatterns (https://github.com/UMCUGenetics/MutationalPatterns) (68) version 3.0.1 was used. For organoids, the R package MutationalPatterns version 3.0.1 was used to analyze SNVs and indels. To compare profiles against COSMIC mutational signatures, the cosine similarity measure was used; version 3.0 was used to analyze mouse tumors, and version 3.2 was used to analyze human organoids. For the indel analysis, indel mutational profiles in the 83-mutation type format were created. For the SNV analysis, SNV mutational profiles in the 6-mutation type and 96-mutation type formats were created. In the indel 83-mutation type format, mutations are divided into 83 categories. Indels are categorized based on several criteria, including size of the indel, nucleotides affected, and the presence of the indel in a repetitive region and/or microhomology region. In the SNV 6-mutation type format, mutations are divided into 6 simple categories as follows: C > A, C > G, C > T, T > A, T > C, and T > G. Additionally, C > T mutations are further subdivided into those that occur within a CpG dinucleotide context and those that do not. In the SNV 96-mutation type format, the 6 mutations outlined above are further subdivided into 16 categories. These categories represent the 16 combinations of nucleotides immediately 5′ and 3′ to each mutated base.

For all of these analyses, samples were pooled into the 4 groups previously defined (see Table 1 and Tumor/normal analysis above). ETBF-specific mutational profiles were created independently with data from the whole-exome sequencing analysis and the whole-genome sequencing analysis by subtracting the sham mutational profiles for each mouse type from the ETBF mutational profile to yield the ETBF-specific mutational profile. The *de novo* ETBF-specific mutational signature was generated by the MutationalPatterns R package using nonnegative matrix factorization (NMF). It was constructed from the *Apc*^min/+^ and *Apc*^min/+^*Msh2^fl/fl^*VC ETBF-specific mutational profiles.

### Statistics.

Summary statistics of data was reported using mean with its standard error. All error bars shown represent standard error of the mean. Mann-Whitney U tests were used to compare the differences in tumor mutation frequency between groups. A *P* value of <0.05 was considered to indicate statistical significance. However, due to the small sample sizes, the tests were primarily exploratory, and no multiplicity adjustments were considered.

### Study approval.

All mice were bred and maintained in a specific pathogen-free barrier facility. Both male and female mice were used, and mice of different genotypes were cohoused during experiments. The Johns Hopkins University Animal Care and Use Committee (MO20M85) and the Bloomington Institutional Animal Care and Use Committee (19-024) approved all experimental protocols.

### Data availability.

The complete experimental data set for the mouse tumor and normal samples was deposited in the Sequence Read Archive (SRA) database under accession number PRJNA643822. The complete experimental data set for the organoid samples was deposited in the European Genome-Phenome Archive (https://ega-archive.org) (under accession number EGAD00001008687).
