# Supplementary material for: Colon Tumors in Enterotoxigenic Bacteroides fragilis (ETBF)-Colonized Mice Do Not Display a Unique Mutational Signature but Instead Possess Host-Dependent Alterations in the APC Gene
Source: Microbiol Spectr. 2022 May 19;10(3):e01055-22. doi: 10.1128/spectrum.01055-22 (PMC9241831; doi:10.1128/spectrum.01055-22)
Supplement: SUPPLEMENTAL FILE 2 — Supplemental material. Download spectrum.01055-21-s002.pdf, PDF file, 6.9 MB [file spectrum.01055-22-s002.pdf]

Supplemental Material

A.)

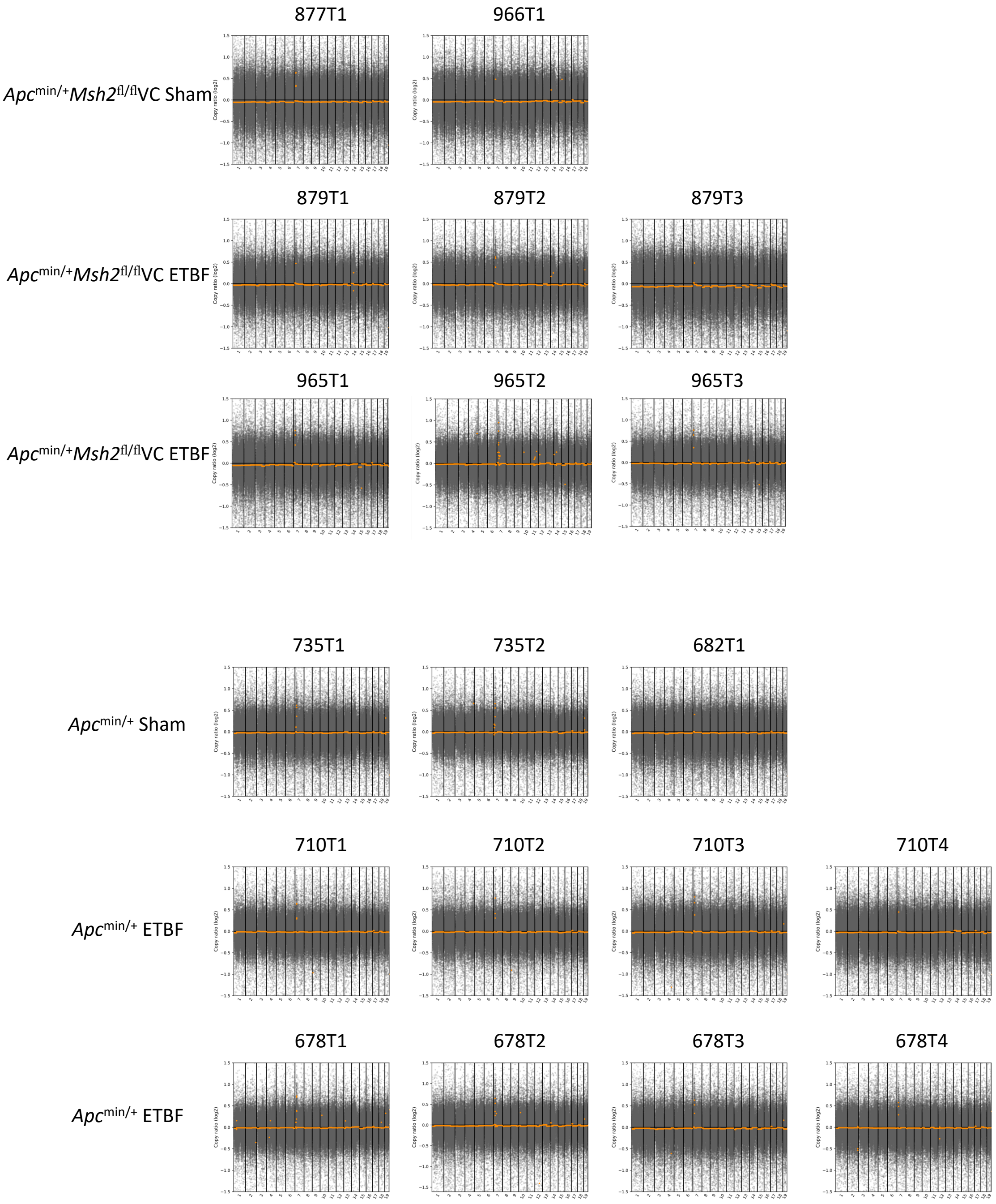

B.)

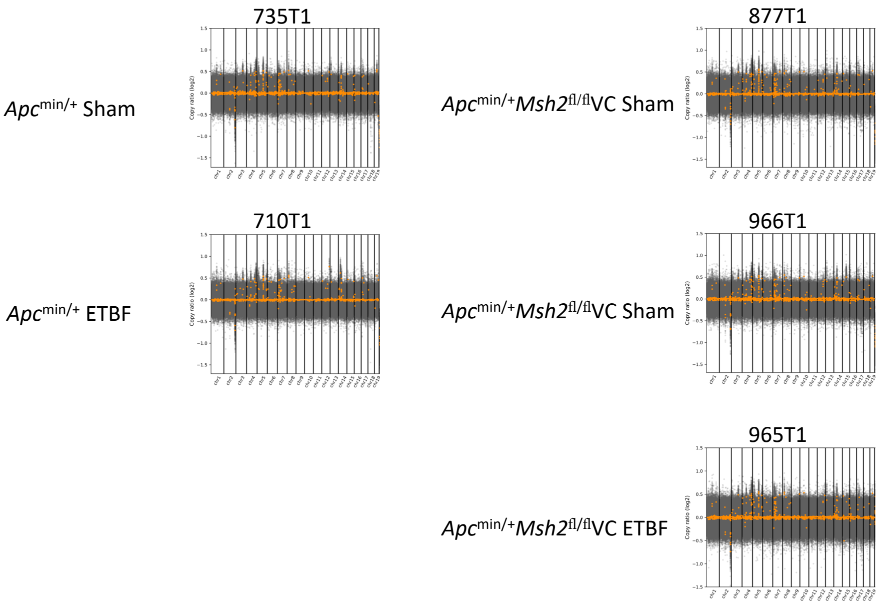

Figure S1: Copy number alterations on each autosomal chromosome are seen sparingly across the genome for both sham-inoculated and ETBF-colonized tumor samples

CNVkit was used to measure copy number alterations. Each gray dot represents an individual data point and the orange lines/dot represent the average copy number variation over a given region. The vertical axis is log2 copy ratio. A log2 copy ratio of 0 represents no difference in copy number at that particular location between the tumor sample and a normal sample from the same mouse. The horizontal axis displays the results from chromosome 1 (left) to chromosome 19 (right). **A.)** Copy number alteration in samples analyzed via whole exome sequencing. **B.)** Copy number alterations in samples analyzed via whole genome sequencing. All data are shown as results for individual tumors from mouse strains as labeled above each figure (mouse number, tumor (T) number).

A.)

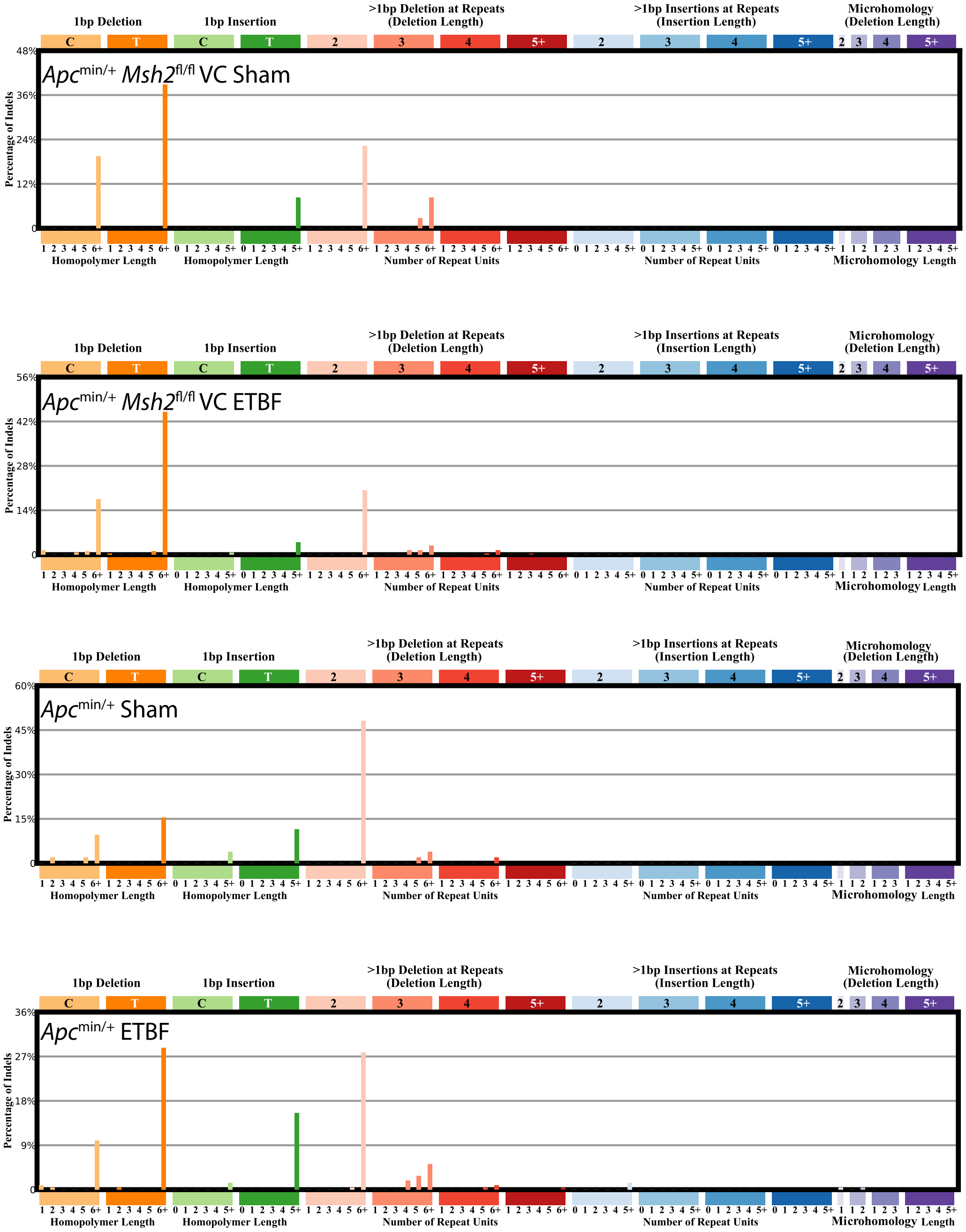
1bp Deletion
1bp Insertion
>1bp Deletion at Repeats (Deletion Length)
>1bp Insertions at Repeats (Insertion Length)
Microhomology (Deletion Length)
Apcmin/+ Msh2fl/fl VC Sham
Apcmin/+ Msh2fl/fl VC ETBF
Apcmin/+ Sham
Apcmin/+ ETBF
Percentage of Indels
Homopolymer Length
Number of Repeat Units
Microhomology Length

B.)

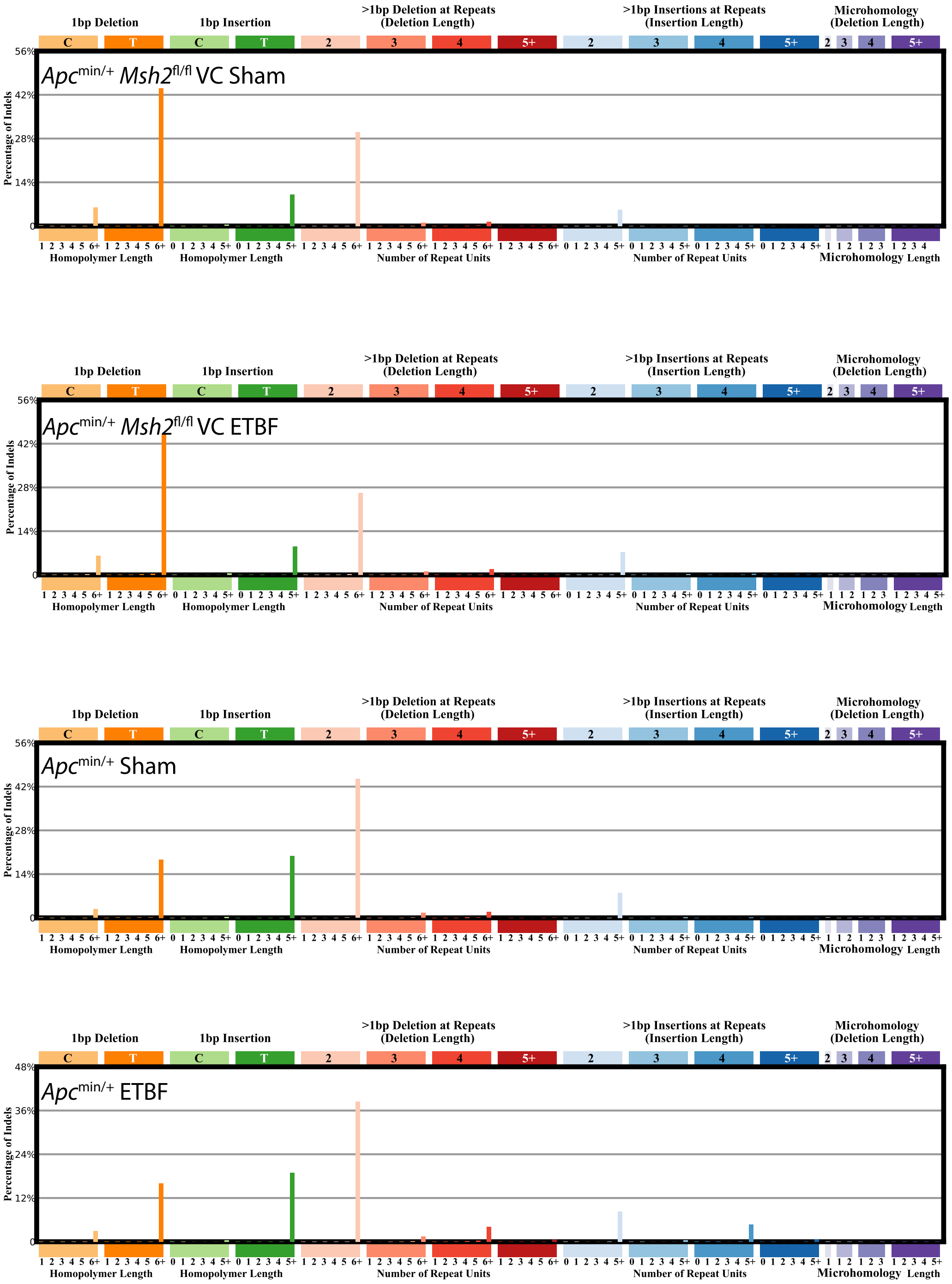
1bp Deletion
1bp Insertion
>1bp Deletion at Repeats (Deletion Length)
>1bp Insertions at Repeats (Insertion Length)
Microhomology (Deletion Length)
Percentage of Indels
Apcmin/+ Msh2fl/fl VC Sham
Apcmin/+ Msh2fl/fl VC ETBF
Apcmin/+ Sham
Apcmin/+ ETBF
Homopolymer Length
Number of Repeat Units
Microhomology Length

Figure S2: Mutational profiles of Insertions/deletions (Indels) show no differences between groups

The R package SigProfilerMatrixGenerator was used to create Indel mutational profiles. Indel mutational profiles are presented in the 83-mutation type format. This format groups Indels based on several criteria including size of the indel, nucleotides affected and the presence of the indel in a repetitive region and/or microhomology region. Note, Y-axis differs between samples. **A.)** Indel mutational profile of whole exome sequencing data in the 83-mutation type format. **B.)** Indel mutational profile of whole genome sequencing data in the 83-mutation type format.

A.)

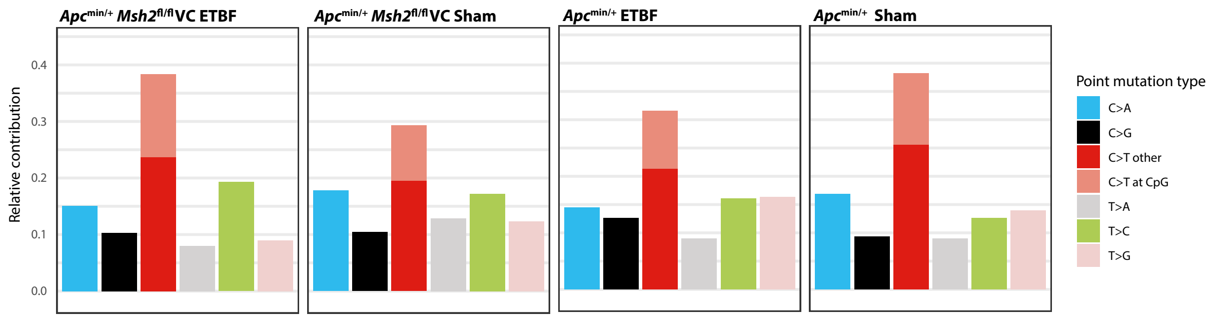
Apcmin/+ Msh2fl/fl VC ETBF
Apcmin/+ Msh2fl/fl VC Sham
Apcmin/+ ETBF
Apcmin/+ Sham
Relative contribution
0.0
0.1
0.2
0.3
0.4
Point mutation type
C>A
C>G
C>T other
C>T at CpG
T>A
T>C
T>G

B.)

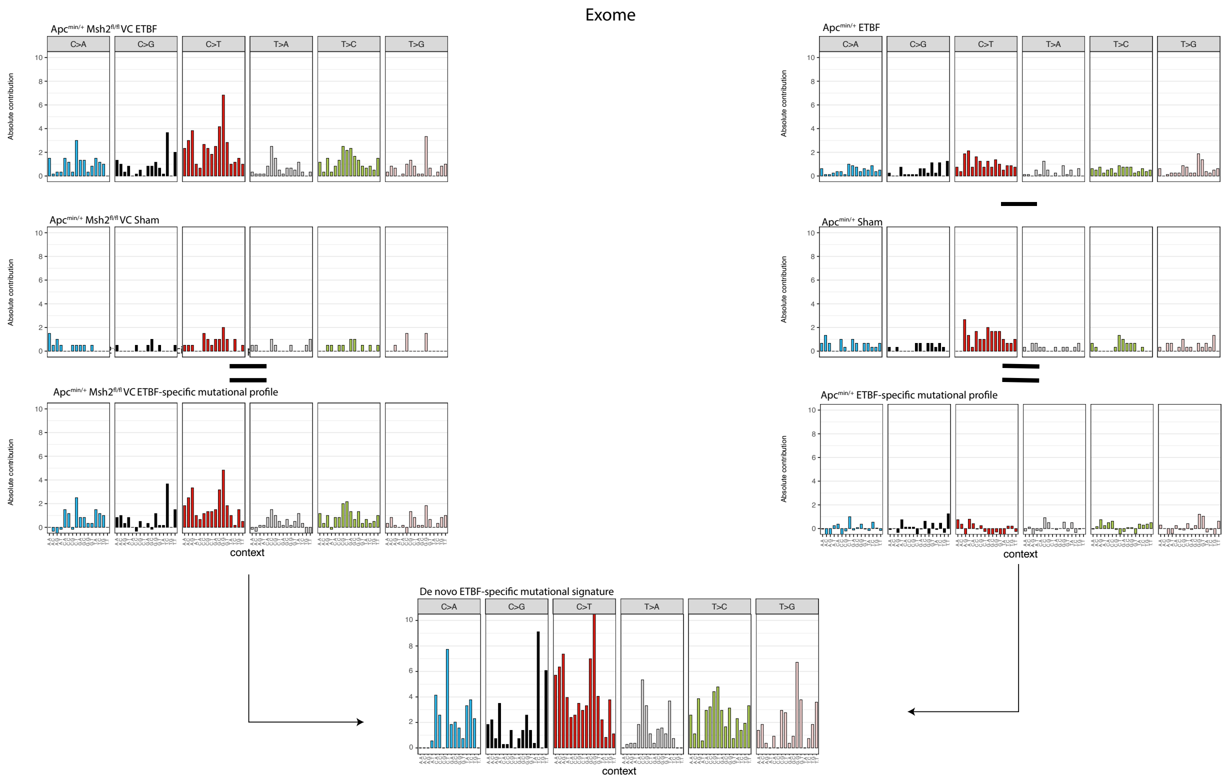
Exome
Apcmin/+ Msh2fl/fl VC ETBF
Apcmin/+ ETBF
Apcmin/+ Msh2fl/fl VC Sham
Apcmin/+ Sham
Apcmin/+ Msh2fl/fl VC ETBF-specific mutational profile
Apcmin/+ ETBF-specific mutational profile
De novo ETBF-specific mutational signature
C>A
C>G
C>T
T>A
T>C
T>G
Absolute contribution
context

C.)

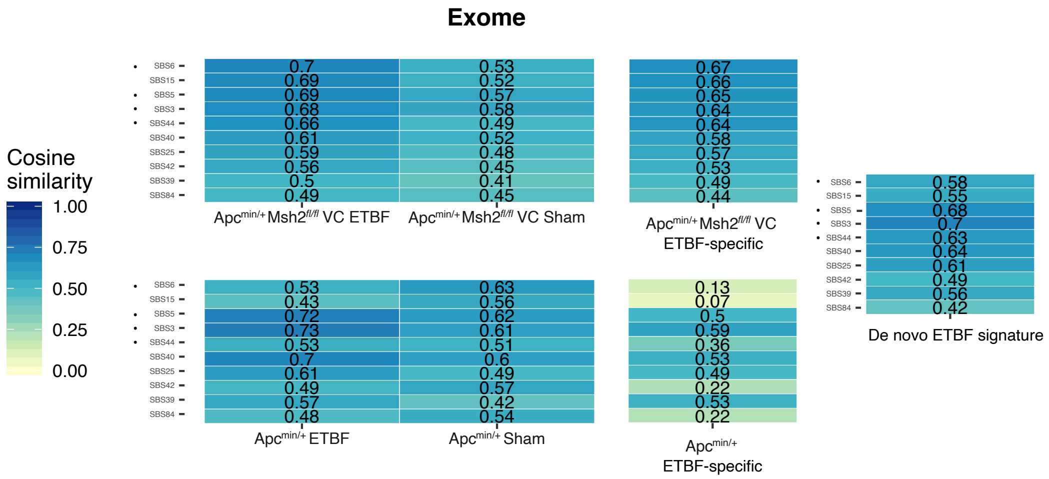

Figure S3: ETBF-specific single base substitution (SBS) mutational profiles extracted from exome sequencing data

The R package MutationalPatterns was used to create SBS mutational profiles. **A.)** SBS mutational profile for whole exome sequencing data in the 6-mutation type format. In the 6-mutation type format, mutations are divided into 6 simple categories: C > A, C > G, C > T, T > A, T > C, T > G. Additionally, C > T mutations are further subdivided into those that occur within a CpG dinucleotide context, and those that do not. **B.)** Graphic detailing how the ETBF-specific mutational profiles were created from the whole exome sequencing data in the 96-mutation type format. In the 96-mutation type format, the 6 mutations outlined above are further subdivided into 16 categories. These categories represent the 16 combinations of nucleotides immediately 5' and 3' to each mutated base. The total number of mutations belonging to each trinucleotide mutation type is presented. **C.)** Heatmaps comparing SBS COSMIC signatures (vertical axis) to the mutational profiles created from whole exome sequencing data in *Apc*$^{min/+}$ mice and *Apc*$^{min/+}$*Msh2*$^{fl/fl}$VC mice. Numbers displayed represent "cosine similarity", which is a metric used to quantify the similarity between any two mutational matrices. Only the top 10

COSMIC SBS signatures are shown. Dots indicate mutational profiles most similar to ETBF signature across multiple analyses.

A.)

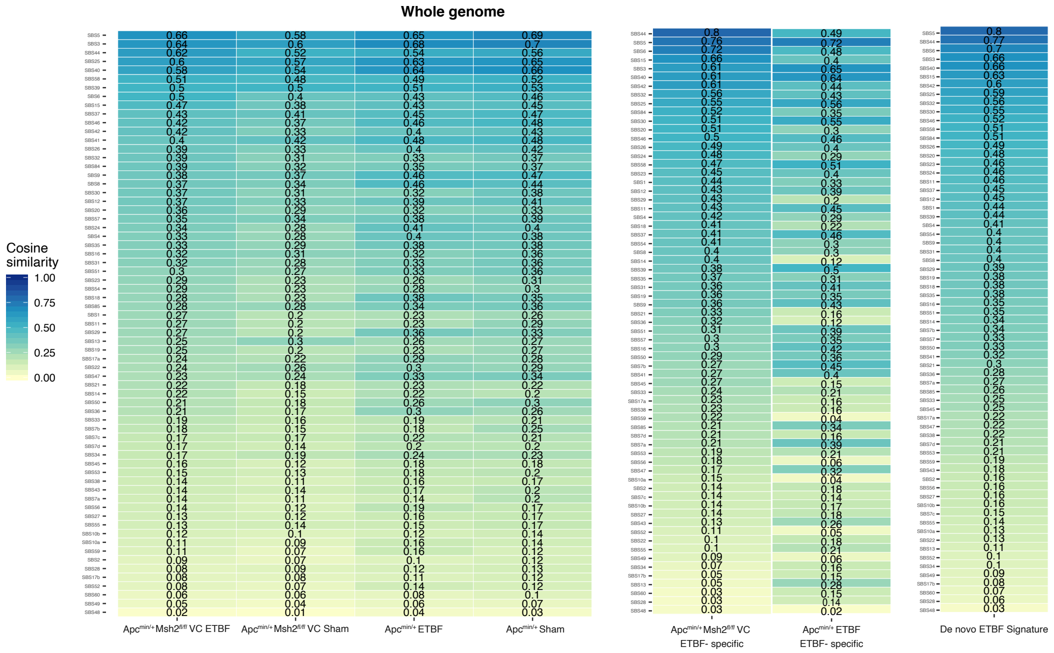

Whole genome
Cosine similarity
1.00
0.75
0.50
0.25
0.00
Apc^min/+Msh2^fl/fl VC ETBF
Apc^min/+Msh2^fl/fl VC Sham
Apc^min/+ ETBF
Apc^min/+ Sham
Apc^min/+Msh2^fl/fl VC ETBF- specific
Apc^min/+ ETBF ETBF- specific
De novo ETBF Signature

B.)

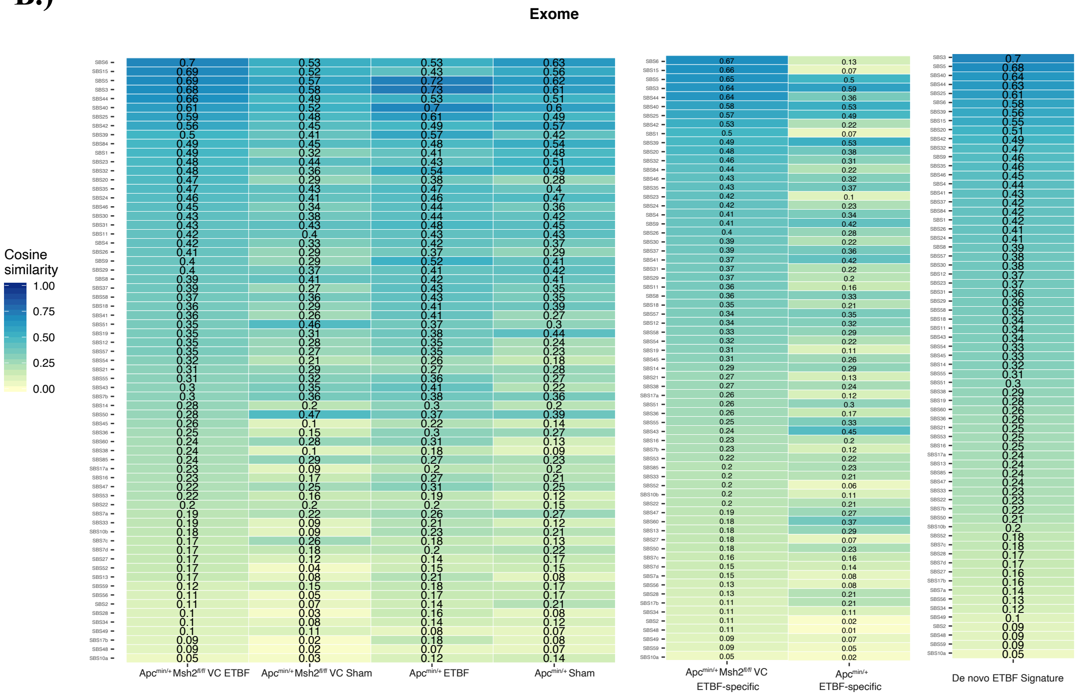

Exome
Cosine similarity
1.00
0.75
0.50
0.25
0.00
Apc^min/+Msh2^fl/fl VC ETBF
Apc^min/+Msh2^fl/fl VC Sham
Apc^min/+ ETBF
Apc^min/+ Sham
Apc^min/+Msh2^fl/fl VC ETBF-specific
Apc^min/+ ETBF-specific
De novo ETBF Signature

C.)

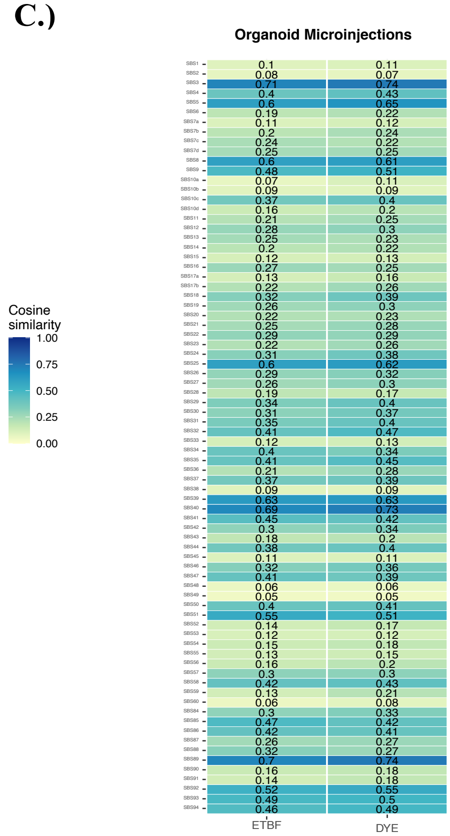

Figure S4: Mutational profiles compared to COSMIC single base substitution (SBS) signatures

Heatmaps comparing SBS COSMIC signatures (vertical axis) to the ETBF-specific mutational profiles created from tumors from Sham mice, tumors from ETBF-colonized mice, the ETBF-specific mutational profile, and the *de novo* ETBF-specific signatures. Numbers displayed represent "cosine similarity", which is a metric used to quantify the similarity between any two mutational matrices. All COSMIC SBS signatures are shown. **A.)** Whole genome sequencing data in *Apc*$^{min/+}$ mice and *Apc*$^{min/+}$*Msh2*$^{fl/fl}$VC mice. **B.)** Exome sequencing data in *Apc*$^{min/+}$ mice and *Apc*$^{min/+}$*Msh2*$^{fl/fl}$VC mice. **C.)** Whole genome sequencing data from human organoids.
